# The relationship among multiple patient-reported outcomes measures for patients with ulcerative colitis receiving treatment with MMX^®^ formulated delayed-release mesalamine

**DOI:** 10.1007/s11136-014-0797-2

**Published:** 2014-09-06

**Authors:** Aaron Yarlas, Linnette Yen, Paul Hodgkins

**Affiliations:** 1 Life Sciences, Optum, 24 Albion Road, Building 400, Lincoln, RI 02865 USA; 2 Global Health Economics and Outcomes Research, Shire Development LLC, Wayne, PA USA; 3 Health Economics and Outcomes Research, Vertex Pharmaceuticals, Cambridge, MA USA

**Keywords:** Delayed-release mesalamine, MMX^®^ mesalamine, Ulcerative colitis, Work productivity, Health-related quality of life

## Abstract

**Purpose:**

Ulcerative colitis (UC) is associated with impaired health-related quality of life (HRQL) and work-related outcomes (WRO). This analysis examined correspondences among measures of HRQL and WRO in patients with UC, as well as the magnitude of each measure’s responsiveness to disease activity and treatment.

**Methods:**

An open-label, prospective trial of delayed-release mesalamine tablets formulated with MMX^®^ technology included 8 weeks of treatment for patients with active mild-to-moderate UC (*n* = 137) and 12 months of maintenance treatment for patients with quiescent UC (*n* = 206). Spearman correlations (*ρ*) measured inter-domain associations across measures of generic HRQL [12-item Short-Form Health Survey (SF-12v2)], disease-specific HRQL [Short Inflammatory Bowel Disease Questionnaire (SIBDQ)], and disease-specific WRO [Work Productivity and Activity Impairment for Specific Health Problems (WPAI:SHP)]. Responsiveness to disease activity and treatment was assessed for each instrument.

**Results:**

Changes in scores from baseline to week 8 were moderately correlated across all instrument domains: 65 of 80 (81 %) between-instrument inter-domain correlations were of moderate magnitude (0.30 < *ρ* < 0.70), with an average magnitude of 0.42 [95 % confidence interval (CI) 0.38–0.46]. Associations between symptom measures were stronger for SIBDQ (|average *ρ*| = 0.41; 95 % CI 0.34–0.48) and WPAI:SHP (0.40; 0.30–0.47) than SF-12v2 (0.30; 0.27–0.34). SIBDQ was most sensitive to treatment [effect size (*d*_*z*_) for change from baseline to week 8 = 0.62; 95 % CI 0.35–0.89], followed by WPAI:SHP (*d*_*z*_ = 0.43; 0.32–0.54) and SF-12v2 (*d*_*z*_ = 0.33; 0.27–0.39).

**Conclusion:**

While the SIBDQ showed the greatest overall responsiveness to disease activity and treatment, all three patient-reported outcomes instruments provided complementary interpretive information regarding the impact of UC treatment.

## Introduction

Ulcerative colitis (UC), an inflammatory bowel disease (IBD), is marked by chronic inflammation of the large intestine and rectum. Symptoms associated with UC include fatigue, a constant urge to defecate, nausea, diarrhea, rectal bleeding, and abdominal pain. The frequency and severity of these symptoms are closely linked to impairments in patient-reported outcomes (PRO), including health-related quality of life (HRQL) [1–7], and work-related outcomes (WRO) such as increased rates of absenteeism and work disability and decreased work productivity [8–16].

Previous research on patients with UC shows improvements in HRQL [17–22] and WRO [18, 23] following treatment when accompanied by decreases in disease activity. For example, both Irvine et al. [20] and Reinisch et al. [23] reported that patients with UC who demonstrated clinical response following treatment had significantly better scores on generic and disease-specific measures of HRQL [the 36-item Short-Form health outcomes survey (SF-36) and the Inflammatory Bowel Disease Questionnaire (IBDQ), respectively] than non-responders. Furthermore, Reinisch et al. [23] found that clinical remission predicted significantly greater improvements in work attendance, and work productivity, and a decreased likelihood of receiving disability benefits.

Cross-sectional studies of patients with UC have typically found concordance between generic and disease-specific HRQL [2, 23–25]. A cross-sectional study by Bernklev et al. [26] that examined the simultaneous relations among generic and disease-specific HRQL and WRO found that both IBDQ and SF-36 scores predicted absenteeism and work disability payments. Cross-sectional studies by Cohen et al. [10] and Gibson et al. [11] found that HRQL (SIBDQ, SF-36) and WRO [Work Productivity and Activity Impairment survey (WPAI)] were associated with disease severity and fatigue, respectively, in patients with UC. Given that few studies have captured the simultaneous impact of treatment on disease-specific HRQL, generic HRQL, and WRO for patients with UC, the degree to which these outcomes are interrelated, and the sensitivity and responsiveness of these outcomes to treatment and disease activity have not been fully established.

The current analysis examines associations among PRO instruments measuring generic and disease-specific HRQL [the 12-item Short-Form Health Survey, version 2 (SF-12v2) and the Short IBDQ (SIBDQ), respectively] and disease-specific WRO [the WPAI: Specific Health Problem (WPAI:SHP)] as well as the extent to which these outcomes are negatively associated with disease activity for patients with mild-to-moderate UC who participated in an open-label prospective trial of delayed-release mesalamine tablets formulated with MMX^®^ (Cosmo Technologies Ltd, Wicklow, Ireland) technology (hereafter referred to as delayed-release mesalamine). The objective of the current analysis is to test several hypotheses regarding the interrelation among these PRO measures, their relative sensitivity to treatment, and their relative responsiveness to changes in disease activity for patients with UC in this clinical treatment trial.

## Methods

### Study design

**Fig. 1:**
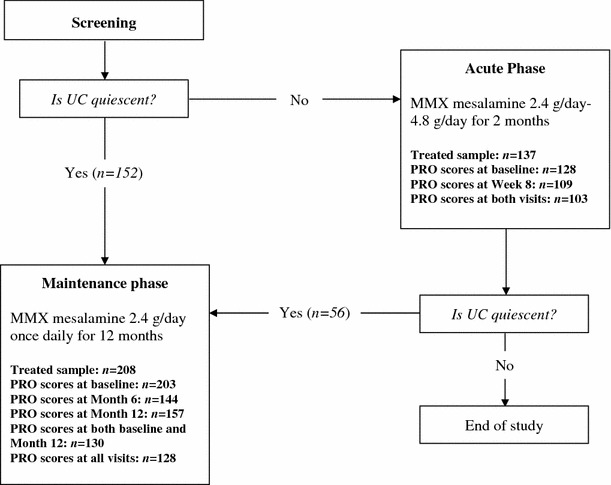
Flowchart of study design. *UC* ulcerative colitis, *MMX* Multi Matrix System, *PRO* patient-reported outcome

Data included in the current analysis were collected from the Strategies in Maintenance for Patients Receiving Long-term Therapy (SIMPLE) study [27], a multicenter, prospective, single-treatment, open-label trial (NCT00446849). This study consisted of a screening period, followed by two phases: an 8-week acute phase, and a 12-month maintenance phase. Figure 1 presents a flowchart of the study design. A more detailed description of the SIMPLE study has been presented elsewhere [27].

Patients diagnosed with mild-to-moderate active UC at screening were entered into the acute phase, where they received daily MMX mesalamine 2.4–4.8 g/day for 8 weeks. Dose titration in increments of 1.2 mg was implemented when necessary throughout the acute phase. Data for all PRO instruments were collected at the acute phase baseline and at the 8-week endpoint.

Patients with quiescent UC at screening, as well as those who achieved quiescence by the acute phase baseline, were able to participate in the 12-month maintenance phase.^1^ In this phase, patients received daily MMX mesalamine 2.4 g/day for 12 months. Data for all PRO instruments were collected from three onsite visits over the 12 months: at baseline, 6, and 12 months (or early withdrawal).

This trial was approved by Institutional Review Boards at each study site. Only patients who provided written informed consent at screening were able to enroll in this trial.

### Outcome measures

#### SF-12v2 (generic HRQL)

The SF-12v2 is a 12-item self-report survey of HRQL with a 4-week recall period [28]. Item responses afford calculation of eight domains representing separate dimensions of functional health and well-being: physical functioning (PF), role physical (RP; role limitations due to physical problems), bodily pain (BP), general health perceptions (GH), vitality (VT), social functioning (SF), role emotional (RE; role limitations due to emotional problems), and mental health (MH). PCS and MCS scores are computed by summing weighted domain scores. SF-12v2 domains and summary scores were standardized using a *T*-score metric (mean = 50, standard deviation = 10) based on a US general population normative sample. Higher scores indicate better health outcomes on all domains and summary scores.

#### SIBDQ (disease-specific HRQL)

The SIBDQ [29] consists of 10 items chosen from among the 32 items on the original IBDQ instrument. This instrument has demonstrated good psychometric properties (i.e., reliability, responsiveness, and construct and criterion validity) in assessment of disease-specific HRQL within the UC patient population [29–32]. The SIBDQ assesses the impact of patients’ IBD symptoms on different aspects of HRQL over the previous 2 weeks, as measured by four domains: bowel symptoms (BS; calculated by summing responses to three items capturing the frequency of abdominal pain, gassiness, and feeling the urge to defecate), systemic symptoms (SS; calculated by summing responses to two items capturing the frequency of fatigue and difficulty in maintaining weight), emotional function (EF; calculated by summing responses to three items capturing the frequency of depression, stress, and anger), and social function (calculated by summing responses to two items capturing the frequency of having to cancel social activities, and being limited in social activities). Responses to each item are also summed to create a total SIBDQ score. Response options for each item range from 1 to 7; thus, possible scores range from 3 to 21 for BS and social function domains, and from 2 to 14 for SS and EF domains, with total scores ranging from 10 to 70. For all domains and the total score, higher scores indicate better health outcomes.

#### WPAI:SHP (WRO)

The WPAI:SHP consists of six items that can be used to measure the impact of a person’s specific health problem (in this case, UC) on WROs, including work time missed, decreases in productivity, and impairment in daily non-work-related activities (e.g., childcare) during the preceding 7 days [33]. The WPAI:SHP has been psychometrically validated within samples of patients with a variety of gastrointestinal disorders, including gastroesophageal reflux disease [34, 35], Crohn’s disease [36], and irritable bowel syndrome [37].

For patients employed over the previous 7 days, four domains were calculated based on item responses: absenteeism (the percentage of work time missed due to a patient’s UC), presenteeism (the percentage of impairment while working due to a patient’s UC relative to their work productivity when healthy), overall work impairment (the percentage of overall work impairment due to a patient’s UC), and activity impairment (the percentage of impairment in non-work activities due to a patient’s UC). Only scores for the activity impairment domain were computed for patients not employed in the previous 7 days. All domain scores range from 0 to 100 %, with lower scores on all domains signifying better WRO (i.e., less impairment).

#### UC symptoms

Two UC symptoms, stool frequency (STF) and rectal bleeding severity (RBS), were measured using single-item patient reports. Measures for each of these symptoms are considered crucial for determining the status of disease in patients with UC, as indicated by their inclusion in two well-established measures of disease activity: the Ulcerative Colitis Disease Activity Index (UC-DAI) [38] and the Mayo score [39]. Previous research has found evidence that STF and RBS items alone are sufficient to estimate disease activity in patients with UC [40].

Patients provided once-daily responses on each via telephone or Internet. For the STF item, patients indicated whether their number of bowel movements that day was the same or only 1 more than their normal frequency (0), 2 or 3 more than their normal frequency (1), or at least 4 more than their normal frequency (2). For the RBS item, patients indicated whether they had no rectal bleeding in their stool (0), streaks of blood in their stool (1), obvious blood in their stool (2), or mostly blood in their stool (3) on the current day. At the time of each on-site visit, the patient’s three most recent responses to each of these items were averaged to create a total score for each symptom.^2^ Lower scores on both measures indicate better outcomes.

### Analysis plan

#### Patient baseline characteristics

Descriptive statistics (i.e., means and standard deviations for continuous variables, frequencies and percentages for categorical variables) were calculated for patient characteristics (e.g., age, gender, and employment status) and values of outcome measures for the full baseline sample of patients in each of the acute and maintenance phases. Descriptive statistics were also calculated separately at maintenance phase baseline for two subsamples of patients in the maintenance phase: those who were identified as quiescent at screening and thus entered the maintenance phase directly (maintenance phase-only subsample), and those who were identified with active disease at screening and thus only entered the maintenance phase after achieving quiescence at the end of the acute phase (acute + maintenance phase subsample).

Baseline values of patient characteristics and outcome scores were compared between maintenance phase-only and acute + maintenance phase subsamples to demonstrate similarity between these subsamples to justify combining both subsamples into a single analysis group. Comparisons of SF-12v2, SIBDQ, and UC symptom scores between these subsamples were conducted in previous analyses of these data [19, 22]; comparisons of patient characteristics and WPAI:SHP scores between the two groups were conducted here. Comparisons between continuous variables were conducted using independent samples *t* tests (two-tailed), while comparisons between categorical variables (gender, employment status) were based on Fisher’s exact test (two-tailed).

#### Correspondence among PRO instruments

The objective of this analytic approach was to estimate the strength of relations among outcomes captured by the three PRO instruments. Analyses falling under this approach were designed to test several hypotheses regarding the relative magnitude of associations among PRO instruments.

Based on previous empirical findings described above, and given the conceptual relatedness among each of these outcomes, Hypothesis 1 was that changes in SF-12v2, SIBDQ, and WPAI:SHP domain scores from baseline to 8-week endpoint during the acute phase would, in general, be moderately correlated (i.e., most correlation coefficients falling within the range of 0.3–0.7).

Since the WPAI:SHP measures a different construct (WRO) than that shared by the other two instruments (HRQL), Hypothesis 2 was that the average inter-domain correlation between the SF-12v2 and the WPAI:SHP would be smaller than the average inter-domain correlation between the SF-12v2 and the SIBDQ.

Also, because the SIBDQ and WPAI:SHP are both designed to capture the impact of disease-specific outcomes, as opposed to generic health outcomes measured by the SF-12v2, Hypothesis 3 was that the average inter-domain correlation between SIBDQ and WPAI:SHP scores would be larger than the average inter-domain correlation between SF-12v2 and WPAI:SHP scores.

To test Hypotheses 1–3, we examined correlations among changes in scores for all domains from each of the three PRO instruments. Change scores for each PRO domain were calculated by subtracting patients’ acute phase baseline score from their acute phase 8-week endpoint score. Spearman correlation coefficients between all change scores were computed to estimate the direction and magnitude of associations.

To estimate the relative strength of associations among each of the PRO instruments, we calculated the average inter-domain correlation between each instrument pair using Fisher’s method [41], which Monte Carlo simulations have shown to produce less biased estimates of mean correlation coefficients [42, 43] the following procedure. First, Spearman coefficients were transformed into *z*-scores using Fisher’s *r*-to-*z* transformation [41] based on the following equation:

1 $$z = \frac{1}{2}\ln \left( {\frac{1 + r}{1 - r}} \right)$$

Next, the average *z*-score was computed as the sum of all *z*-scores divided by the number of *z*-scores. Finally, the average *z*-score was transformed back into the average correlation coefficient using the inverse of Fisher’s *r*-to-*z* transformation, based on the following equation:

2 $$r = \frac{{\exp \left( {2z} \right) - 1}}{{\exp \left( {2z} \right) + 1}}$$

For each correlation coefficient, a 95 % confidence interval (CI) was calculated using the following procedure. First, the correlation coefficient (*ρ*) was transformed into a *z*-score (*z*_*ρ*_) using Fisher’s *r*-to-*z* transformation (Eq. 1). Second, the standard error for *z*_*ρ*_ was calculated using the following equation: [41, 44]

3 $${\text{SE}}_{{z_{\rho } }} = \frac{1}{{\sqrt {n - 3} }}$$

Third, the 95 % CI for *z*_*ρ*_ (95 % ${\text{CI}}_{{z_{\rho } }}$) was calculated by multiplying ${\text{SE}}_{{z_{\rho } }}$ by 1.96. Fourth, the 95 % ${\text{CI}}_{{z_{\rho } }}$ was transformed into the 95 % CI_*ρ*_ using the inverse of Fisher’s *r*-to-*z* transformation (Eq. 2).

#### Responsiveness of PRO instruments to disease activity and sensitivity to treatment

The objective of this analytic approach was to estimate the relative degree to which changes in each of the three PRO instruments corresponded to changes in UC symptoms (i.e., STF and RBS) over the course of treatment. Analyses falling under this approach were designed to test hypotheses regarding the responsiveness among instruments to disease activity and their sensitivity to treatment.

Since both the SIBDQ and WPAI:SHP, but not the SF-12v2, explicitly assess the impact of UC-related symptoms on patient outcomes, Hypothesis 4 was that the correlations between changes in SIBDQ and WPAI:SHP scores and changes in UC symptoms would generally be larger than correlations between changes in SF-12v2 scores and changes in these symptoms.

Given previously established findings from this trial that HRQL was lower for patients who experienced clinical recurrence (based on the recurrence of symptoms) at the 12-month maintenance phase endpoint as compared to non-recurrent patients [19, 22], and following the same logic of the previous hypothesis, Hypothesis 5 was that differences in change scores between recurrent and non-recurrent patients would be relatively larger for the SIBDQ and WPAI:SHP than for the SF-12v2.

Finally, because disease-specific HRQL captures more proximally the impact of treatment on patient outcomes than does generic HRQL or WRO, Hypothesis 6 was that the SIBDQ would exhibit greater sensitivity to acute treatment than would the SF-12v2 or WPAI:SHP.

The responsiveness of HRQL and WRO to disease activity was captured using two analytic approaches. First, the correspondences between changes in PRO domain scores and changes in symptom scores during the acute phase were examined using Spearman correlations. Change scores for symptom measures were calculated by subtracting each patient’s acute phase baseline score from their acute phase 8-week endpoint score. To test the relative strength of associations between the different PRO instruments and the measures of disease activity in Hypothesis 4, we calculated the average correlations across all domain scores within each instrument with scores on each symptom measure using Fisher’s *r*-to-*z* transformation procedure described above.

The responsiveness of each PRO instrument to changes in disease activity was also assessed by comparing PRO domain scores between patients who did or did not exhibit clinical recurrence at the 12-month maintenance phase assessment. Patients were classified as exhibiting clinical recurrence if they reported 4 or more bowel movements per day above their normal frequency and the presence of rectal bleeding, urgency to defecate, or abdominal pain. Univariate analysis of covariance (ANCOVA) models, with recurrence status as a between-subjects’ factor and patients’ age, gender, body mass index (BMI), and maintenance baseline domain value as covariates, statistically compared recurrent and non-recurrent patients on each instrument domain. Cohen’s *d* effect sizes [45] for standardized differences between independent-group estimated means^3^ were calculated for all comparisons to indicate the strength of the effect of classification group for each domain score. Interpretation of these effects followed Cohen’s conventional guidelines for interpretation of magnitude (i.e., small effect size: *d* ≈ 0.2, medium effect size: *d* ≈ 0.5, large effect size: *d* ≈ 0.8) [45].

The sensitivity of each PRO instrument to acute treatment was examined using paired-sample *t* tests to compare mean scores between baseline and 8-week assessments. Magnitude of change was estimated using Cohen’s *d*_*z*_ effect sizes [45] for standardized mean differences across dependent samples.^4^

No imputation techniques were used for patients missing data at a visit; only observed values were analyzed at each time point. Average correlations and effect sizes were calculated using Microsoft Excel (2007; Redmond, WA, USA). All other statistical analyses were performed using SPSS for Windows, version 17.0.2 (2009; Chicago, IL, USA).

## Results

### Patient baseline characteristics

Table 1 presents descriptive statistics for patients’ baseline age, gender, and employment status; domain and summary scores for the SF-12v2, SIBDQ, and WPAI:SHP; UC symptom scores for the full sample of patients in each of the acute and maintenance phases; and the maintenance phase baseline values of the maintenance phase-only and acute + maintenance phase subsamples. Previously published comparisons between these subsamples yielded no statistically significant group differences for either SF-12v2, SIBDQ, or UC symptom scores (all *P* > 0.05) [19, 22]. Subsample comparisons of patient characteristics and WPAI:SHP scores conducted here found no statistically significant differences between the two groups in gender distribution, employment status, or any WPAI:SHP domains (all *P* > 0.05), although a statistically significant difference in age was observed (*P* < 0.05), with patients in the maintenance phase-only subsample being, on average, 5.5 years older than those in the acute + maintenance phase subsample.

**Table 1:** Baseline patient characteristics and SF-12v2, SIBDQ, WPAI:SHP, and UC symptom scores for acute and maintenance phase samples and subsamples

	Acute phase baseline (*n* = 132)	Maintenance phase baseline (*n* = 206)	Maintenance phase baseline for acute + maintenance phase subsample (*n* = 56)	Maintenance phase baseline for maintenance phase-only subsample (*n* = 150)
Age [mean (SD)]	43.4 (14.1)	46.9 (13.7)	42.9 (14.4)	48.4 (13.2)^a^
Female [*n* (%)]	74 (56.1 %)	106 (51.5 %)	27 (48.2 %)	79 (52.7 %)
Employed [*n* (%)]	84 (63.6 %)	150 (72.8 %)	42 (75.0 %)	108 (72.0 %)
SF-12v2 [mean (SD)]				
Physical functioning	48.1 (10.1)	53.5 (6.8)	54.3 (4.4)	53.2 (7.5)
Role physical	44.8 (10.5)	52.8 (6.7)	53.0 (6.1)	52.7 (6.9)
Bodily pain	45.2 (11.3)	53.6 (7.2)	55.1 (6.1)	53.1 (7.4)
General health	44.6 (10.7)	51.8 (7.8)	52.4 (5.9)	51.5 (8.5)
Vitality	47.0 (10.3)	53.6 (8.5)	54.8 (8.8)	53.2 (8.4)
Social functioning	44.7 (12.4)	52.9 (7.3)	53.3 (7.0)	52.8 (7.4)
Role emotional	47.0 (10.7)	51.7 (7.3)	51.8 (7.5)	51.6 (7.2)
Mental health	48.1 (10.8)	53.1 (8.6)	54.3 (8.9)	52.7 (8.4)
Physical summary (PCS)	45.4 (9.8)	53.2 (6.5)	53.9 (4.4)	53.0 (7.1)
Mental summary (MCS)	47.3 (10.1)	52.3 (8.2)	53.0 (8.6)	52.1 (8.0)
SIBDQ [mean (SD)]				
Bowel symptoms	12.8 (4.3)	18.5 (2.6)	18.6 (2.4)	18.4 (2.7)
Systemic symptoms	8.9 (3.0)	10.8 (2.5)	11.2 (2.6)	10.6 (2.6)
Emotional function	14.0 (4.3)	17.3 (3.0)	17.4 (3.3)	17.2 (2.9)
Social function	10.1 (3.4)	13.2 (1.5)	13.2 (1.5)	13.2 (1.5)
Total score	45.9 (12.9)	59.8 (7.7)	60.7 (7.5)	59.4 (7.8)
WPAI [mean (SD)]				
Absenteeism	8.8 (21.1)	0.6 (3.5)	1.4 (5.9)	0.3 (1.7)
Presenteeism	27.5 (26.3)	5.1 (9.7)	5.2 (11.3)	5.1 (9.1)
Overall work impairment	30.0 (29.2)	5.5 (10.8)	6.1 (13.7)	5.3 (9.6)
Activity impairment	35.5 (31.2)	7.6 (14.1)	6.6 (14.4)	8.0 (14.0)
UC symptoms [mean (SD)]				
Stool frequency	0.73 (0.69)	0.15 (0.33)	0.15 (0.38)	0.14 (0.31)
Rectal bleeding severity	0.88 (0.77)	0.02 (0.12)	0.02 (0.11)	0.03 (0.12)

*SF-12v2* 12-item Short-Form Health Survey, version 2, *SIBDQ* Short Inflammatory Bowel Disease Questionnaire, *WPAI:SHP* Work Productivity and Activity Impairment: Specific Health Problem, *UC* ulcerative colitis, *SD* standard deviation

^a^ *P* < 0.05 for differences between acute + maintenance phase and maintenance phase-only subsamples

### Correspondence among changes in PRO domain scores during the acute phase

Spearman coefficients for inter-domain correlations among baseline-endpoint changes in SF-12v2, SIBDQ, and WPAI:SHP domain scores in the acute phase are presented in Table 2. Inter-domain correlations across the three instruments reflect mostly moderate associations, with the absolute values for 65 out of the 80 inter-domain correlation coefficients (81 %) ranging between 0.30 and 0.70 [the absolute values for the remaining 15 inter-domain correlation coefficients were small (≤0.30)], with an average magnitude of 0.42 (95 % CI 0.38–0.46). The magnitude of correlation coefficients between SF-12v2 and SIBDQ domain change scores ranged from 0.24 to 0.70 [with absolute values for 28 of the 32 (87.5 %) coefficients between 0.30 and 0.70, and absolute values for the remaining four coefficients (12.5 %) at 0.30 or below], with an average magnitude of 0.44 (95 % CI 0.39–0.49); the magnitude of correlation coefficients between SF-12v2 and WPAI:SHP domain change scores ranged from 0.07 to 0.57 [with absolute values for 23 of the 32 (71.9 %) coefficients between 0.30 and 0.70, and with absolute values for the remaining nine coefficients (28.1 %) at 0.30 or below], with an average magnitude of 0.37 (0.31–0.42); and magnitude of correlation coefficients between SIBDQ and WPAI:SHP domain change scores ranged from 0.13 to 0.68 [with absolute values for 14 of the 16 (87.5 %) coefficients between 0.30 and 0.70, and absolute values for the remaining two coefficients (12.5 %) at 0.30 or below], with an average magnitude of 0.47 (0.36–0.59).

**Table 2:**
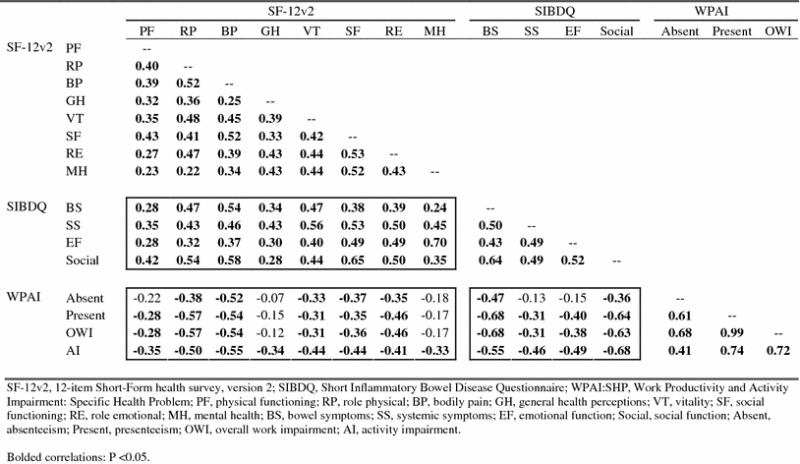
Spearman coefficients for inter-domain correlations among acute phase baseline-endpoint SF-12v2, SIBDQ, and WPAI:SHP change scores

### Responsiveness of PRO instruments to changes in disease activity

Spearman correlation coefficients between acute phase change scores of the PRO domains and symptoms measures are presented in Table 3. The magnitude of correlation coefficients between changes in SF-12v2 domain and changes in symptom measures ranged from 0.15 to 0.45 [with absolute values for 9 of the 16 (56.3 %) coefficients between 0.30 and 0.70, and absolute values for the remaining seven coefficients (43.8 %) at 0.30 or below], with an average magnitude of 0.30 (95 % CI 0.27–0.34); the magnitude of correlation coefficients between changes in SIBDQ and changes in symptom measures ranged from 0.26 to 0.52 [with absolute values for seven of the eight (87.5 %) coefficients between 0.30 and 0.70, and absolute values for the remaining one coefficient (12.5 %) at 0.30 or below], with an average magnitude of 0.41 (0.34–0.48); and the magnitude of correlation coefficients between changes in WPAI:SHP scores and symptom measures ranged from 0.25 to 0.51 [with absolute values for seven of the eight (87.5 %) coefficients between 0.30 and 0.70, and absolute values for the remaining one coefficient (12.5 %) at 0.30 or below], with an average magnitude of 0.40 (0.34–0.47). Also observed was a difference in the strengths of association across the two symptoms: the average magnitude of the correlation among HRQL and WRO domain change scores and changes in STF was 0.33 (0.27–0.36), while the average magnitude among HRQL and WRO domain change scores and changes in RBS was 0.39 (0.35–0.44).

**Table 3:** Spearman coefficients for correlations between acute phase baseline-endpoint SF-12v2, SIBDQ, and WPAI:SHP change scores and UC symptom change scores

		Stool frequency	Rectal bleeding severity
UC symptoms	Stool frequency	–	–
	Rectal bleeding severity	**0.52 (0.36, 0.65)**	–
SF-12v2	Physical functioning	**−0.25 (−0.42, −0.05)**	**−0.30 (−0.47, −0.12)**
	Role physical	**−0.24 (−0.42, −0.05)**	**−0.30 (−0.47, −0.12)**
	Bodily pain	**−0.33 (−0.49, −0.15)**	**−0.33 (−0.50, −0.15)**
	General health	**−0.31 (−0.47, −0.12)**	**−0.28 (−0.45, −0.09)**
	Vitality	**−0.29 (−0.46, −0.11)**	**−0.45 (−0.59, −0.28)**
	Social functioning	**−0.34 (−0.50, −0.15)**	**−0.40 (−0.55, −0.23)**
	Role emotional	**−0.24 (−0.41, −0.05)**	**−0.35 (−0.51, −0.16)**
	Mental health	−0.15 (−0.33, 0.05)	**−0.26 (−0.44, −0.07)**
SIBDQ	Bowel symptoms	**−0.43 (−0.58, −0.26)**	**−0.52 (−0.65, −0.37)**
	Systemic symptoms	**−0.35 (−0.51, −0.17)**	**−0.40 (−0.55, −0.22)**
	Emotional function	**−0.26 (−0.43, −0.07)**	**−0.41 (−0.56, −0.24)**
	Social function	**−0.37 (−0.52, −0.18)**	**−0.50 (−0.63, −0.34)**
WPAI:SHP	Absenteeism	**0.25 (0.01, 0.46)**	**0.38 (0.15, 0.57)**
	Presenteeism	**0.38 (0.16, 0.57)**	**0.42 (0.20, 0.60)**
	Overall work impairment	**0.36 (0.13, 0.55)**	**0.42 (0.20, 0.60)**
	Activity impairment	**0.50 (0.33, 0.63)**	**0.51 (0.35, 0.64)**

Lower and upper boundary values for 95 % CIs around correlation coefficients are in parenthesis

*SF-12v2* 12-item Short-Form Health Survey, version 2, *SIBDQ* Short Inflammatory Bowel Disease Questionnaire, *WPAI:SHP* Work Productivity and Activity Impairment: Specific Health Problem

Bolded correlations are statistically different from 0 (*P* < 0.05)

Table 4 presents month 12 estimated mean SF-12v2, SIBDQ, and WPAI:SHP domain scores (adjusted for patients’ age, gender, BMI, and maintenance baseline value) between patients who did or did not exhibit clinical recurrence at the end of the maintenance phase. The majority of domains (i.e., all but the PF domain on the SF-12v2, the SS domain on the SIBDQ, and the absenteeism domain on the WPAI:SHP) indicated significantly worse outcomes for patients with recurrent symptoms, *P* < 0.05 for all differences. Effect sizes for SF-12v2 domains were small to moderate, ranging from 0.30 (PF) to 0.60 (BP) with an average effect size of 0.45 (95 % CI 0.39–0.51). Average effect sizes for SIBDQ and WPAI:SHP were negatively impacted by each having one domain showing negligible group effects (*d* = 0.03 for the SIBDQ SS domain, *d* = −0.06 for the WPAI:SHP absenteeism domain); mean effect sizes across SIBDQ domains were 0.48 (0.14–0.82), ranging from 0.03 (SS) to 0.86 (BS); and across WPAI:SHP domains were 0.41 (0.08–0.73), ranging from −0.06 (absenteeism) to 0.71 (presenteeism).

**Table 4:** Comparison of estimated mean SF-12v2, SIBDQ, and WPAI:SHP Scores (adjusted for age, gender, BMI, and baseline value) at 12-month maintenance phase endpoint for patients with clinically recurrent or non-recurrent symptoms

	Estimated mean (SE) for non-recurrent patients (*n* = 117)	Estimated mean (SE) for recurrent patients (*n* = 29)	*P* ^a^	Effect size (*d*)
SF-12v2				
Physical functioning	53.8 (0.60)	51.3 (1.23)	ns	0.30
Role physical	53.1 (0.66)	49.3 (1.34)	<0.05	0.42
Bodily pain	53.5 (0.76)	47.4 (1.53)	<0.001	0.60
General health	53.2 (0.71)	48.8 (1.43)	<0.01	0.46
Vitality	53.9 (0.75)	48.6 (1.53)	<0.01	0.52
Social functioning	52.8 (0.75)	48.6 (1.51)	<0.05	0.41
Role emotional	52.8 (0.73)	49.0 (1.47)	<0.05	0.39
Mental health	53.6 (0.70)	49.0 (1.43)	<0.01	0.49
SIBDQ				
Bowel symptoms	18.3 (0.28)	15.1 (0.58)	<0.001	0.86
Systemic symptoms	10.9 (0.21)	11.0 (0.43)	ns	0.03
Emotional function	17.6 (0.31)	15.8 (0.62)	<0.01	0.46
Social function	13.2 (0.18)	11.8 (0.37)	<0.001	0.58
WPAI:SHP				
Absenteeism	2.4 (1.27)	1.5 (2.62)	ns	−0.06
Presenteeism	3.9 (1.70)	16.6 (3.34)	<0.01	0.71
Overall work impairment	6.1 (2.10)	18.6 (4.35)	<0.05	0.55
Activity impairment	7.5 (1.66)	16.7 (3.29)	<0.05	0.43

*SF-12v2* 12-item Short-Form Health Survey, version 2, *SIBDQ* Short Inflammatory Bowel Disease Questionnaire, *WPAI:SHP* Work Productivity and Activity Impairment: Specific Health Problem, *BMI* body mass index, *SE* standard error of the estimated mean, *ns* not statistically significant

^a^ *P* values for comparison of means as a function of recurrence status were derived from analysis of covariance models with recurrence status as a between-subjects factor, and patients’ age, gender, BMI, and baseline value on that domain as covariates

### Sensitivity of SF-12v2, SIBDQ, and WPAI:SHP to acute treatment

Table 5 presents mean SF-12v2, SIBDQ, and WPAI:SHP domain and summary scores at acute phase baseline and 8-week endpoint from patients who completed these measures at both times. Comparison of scores between visits using paired-sample *t* tests revealed statistically significant improvements (*P* < 0.05 for all differences) for 18 of the 19 domains and summary measures, with only the RE domain of the SF12v2 showing no significant change over time.

**Table 5:** Comparison of Mean SF-12v2, SIBDQ, WPAI:SHP, and UC symptom scores from baseline to week 8 of the acute phase

	*N*	Baseline [mean (SE)]	Week 8 [mean (SE)]	Mean difference	*P* ^a^	Effect size (*d*_*z*_)
SF-12v2						
Physical functioning	107	49.1 (0.88)	51.2 (0.76)	2.1	<0.01	0.27
Role physical	107	45.8 (0.95)	49.4 (0.85)	3.6	<0.001	0.40
Bodily pain	107	46.5 (1.01)	51.1 (0.95)	4.6	<0.001	0.45
General health	107	45.3 (1.03)	48.6 (0.91)	3.3	<0.01	0.32
Vitality	107	47.7 (0.99)	51.8 (0.90)	4.1	<0.001	0.38
Social functioning	107	45.7 (1.13)	49.4 (0.98)	3.7	<0.01	0.31
Role emotional	107	48.3 (0.90)	49.8 (0.93)	1.5	ns	0.15
Mental health	107	49.0 (0.97)	52.5 (0.93)	3.5	<0.001	0.36
PCS	107	46.4 (0.89)	49.8 (0.81)	3.4	<0.001	0.45
MCS	107	48.2 (0.91)	51.1 (0.88)	2.9	<0.01	0.29
SIBDQ						
Bowel symptoms	103	13.2 (0.43)	17.2 (0.35)	4.0	<0.001	1.03
Systemic symptoms	103	9.2 (0.3)	10.6 (0.26)	1.4	<0.001	0.47
Emotional function	103	14.6 (0.41)	16.4 (0.37)	1.8	<0.001	0.44
Social function	103	10.5 (0.32)	12.1 (0.27)	1.6	<0.001	0.55
Total score	103	47.5 (1.24)	56.3 (1.05)	8.8	<0.001	0.79
WPAI:SHP						
Absenteeism	70	6.8 (2.18)	2.8 (1.54)	–4.0	<0.05	0.26
Presenteeism	69	25.2 (2.95)	12.9 (2.59)	–12.3	<0.001	0.46
Work productivity	69	27.4 (3.25)	13.6 (2.71)	–13.8	<0.001	0.47
Activity impairment	103	32.4 (2.97)	18.2 (2.54)	–14.2	<0.001	0.52

*SF-12v2* 12-item Short-Form Health Survey, version 2, *SIBDQ* Short Inflammatory Bowel Disease Questionnaire, *WPAI:SHP* Work Productivity and Activity Impairment: Specific Health Problem, *BMI* body mass index, *SE* standard error of the mean, *ns* not statistically significant

^a^ *P* values for comparison of means between visits were derived from paired-sample *t* tests with a two-tailed test for statistical significance

Examination of Cohen’s *d*_*z*_ effect sizes for standardized mean differences yielded different patterns of magnitude in changes across the three instruments. Effect sizes for changes in SIBDQ domains [which ranged from moderate (0.44 for EF) to large (1.03 for BS); mean *d*_*z*_ = 0.62, 95 % CI 0.35–0.89] were generally larger than those observed for SF-12v2 domains [which ranged from small (0.15 for RE) to moderate (0.45 for BP); mean *d*_*z*_ = 0.33, 95 % CI 0.27–0.39] and for WPAI:SHP domains [which ranged from small (0.26 for absenteeism) to moderate (0.52 for activity impairment); mean *d*_*z*_ = 0.43, 95 % CI 0.32–0.54].

## Discussion

Findings from the current study provide several pieces of evidence regarding the correspondence among instruments measuring different PROs, and between each of these PRO instruments with measures of disease activity. Table 6 summarizes each of the six hypotheses tested in this analysis, as well as whether the findings were supportive or non-supportive of the hypothesized relationships among variables.

**Table 6:** Summary of hypotheses tested

Number	Statement of hypothesis	Reasoning underlying hypothesis	Hypothesis supported by findings?
1	Most inter-domain correlations between PRO instruments will be moderately sized (i.e., falling within the range of 0.30–0.70)	Findings from prior research	*Yes*; 65 of 80 (81 %) of inter-domain correlations were within this range
2	Correlations between SF-12v2 and SIBDQ domains will be larger than between SF-12v2 and WPAI:SHP domains	The SF-12v2 and SIBDQ measure the same underlying construct (HRQL), while the WPAI:SHP measures a different construct (WRO)	*Yes*; the magnitude of the average inter-domain correlation between SF-12v2 and SIBDQ (0.44) was higher than between SF-12v2 and WPAI:SHP (−0.37)
3	Correlations between SIBDQ and WPAI:SHP domains will be larger than between SF-12v2 and WPAI:SHP domains	The SIBDQ and WPAI:SHP measure UC-specific health outcomes, while the SF-12v2 measures generic health outcomes	*Yes*; the magnitude of the average inter-domain correlation between SIBDQ and WPAI:SHP (0.47) was higher than between SF-12v2 and WPAI:SHP (0.37)
4	Changes in UC symptoms from baseline to week 8 will correlate more highly with SIBDQ and WPAI:SHP domains than with SF-12v2 domains	Because the SIBDQ and WPAI:SHP measure UC-specific health outcomes, while the SF-12v2 measures generic health outcomes, the former two instruments should be more responsive to changes in UC-specific symptoms	*Yes*; the magnitude of the average correlations of UC symptom scores with domains of the SIBDQ (0.41) and WPAI:SHP (0.40) was higher than between UC symptom scores and SF-12v2 domains (0.30)
5	Differences in change scores as a function of month 12 clinical recurrence status will be larger for SIBDQ and WPAI:SHP domains than for SF-12v2 domains	Because the SIBDQ and WPAI:SHP measure UC-specific health outcomes, while the SF-12v2 measures generic health outcomes, the former two instruments should be more responsive to changes in UC-specific symptoms	*No*; the magnitude of average effect sizes for differences in domain scores between clinical recurrence status groups was similar across all PRO instruments (0.48 for SIBDQ, 0.45 for SF-12v2, and 0.44 for the WPAI:SHP)
6	SIBDQ and WPAI:SHP domains will show larger treatment effects during the acute treatment phase than will SF-12v2 domains	Because the SIBDQ and WPAI:SHP measure UC-specific health outcomes, while the SF-12v2 measures generic health outcomes, the former two instruments should be more sensitive to treatment that decreases UC-specific symptoms	*Yes*; the magnitude of average effect size for changes in scores from Baseline to week 8 was larger for domains of the SIBDQ (average *d* = 0.62) and WPAI:SHP (0.43) than for SF-12v2 domains (0.33)

Consistent with our initial hypothesis, inter-domain correlations for acute phase baseline-endpoint change scores across SF-12v2, SIBDQ, and WPAI:SHP instruments mostly ranged from 0.30 to 0.70, indicating generally moderate concordance in the improvement of each outcome over time. The consistency in scores across instruments also emerged from comparisons of scores following treatment, with all but one domain (RE on the SF-12v2) showing statistically significant improvement from acute phase baseline to 8-week endpoint. Finally, domains from all three of these instruments showed improvement with decreases in stool frequency and rectal bleeding during the acute phase, and all instruments were generally sensitive to patient recurrent status at the maintenance phase endpoint.

While the central results of this analysis indicated close correspondence among patient outcomes, several differences in their associations emerged that were consistent with our hypotheses. Our second and third hypotheses, which predicted that the association between SF-12v2 and WPAI:SHP scores would be weaker than the associations between SF-12v2 and SIBDQ scores (Hypothesis 2) and weaker than associations between SIBDQ and WPAI:SHP scores (Hypothesis 3), were both supported by the data. Specifically, the average correlation coefficient between changes in scores on the SF-12v2 and WPAI:SHP domains from baseline to the 8-week endpoint in the acute phase was smaller than for average correlations of change scores across domains for either of the other two pairings.

Given that the SIBDQ, but not the SF-12v2, explicitly probes the impact of symptoms on patients’ perceptions of HRQL, we hypothesized that the SIBDQ would show greater sensitivity to disease activity than the SF-12v2, as indicated by stronger correlations with UC symptom scores (Hypothesis 4) and better discrimination between patients with clinically recurrent and non-recurrent status (Hypothesis 5). The observed results supported Hypothesis 4: The magnitude of the average correlation coefficient between UC symptom measures and SIBDQ domains (0.41, 95 % CI 0.34–0.48) was approximately 0.11 larger than that between symptoms and SF-12v2 domains (0.30, 0.27–0.34). Results were not supportive of Hypothesis 5: The average effect sizes for standardized mean differences between recurrent and non-recurrent patients were of similar magnitude for domain scores of the SIBDQ (average *d* = 0.48, 95 % CI 0.14–0.82), the SF-12v2 (average *d* = 0.45, 0.39–0.51), and the WPAI:SHP (*d* = 0.41, 0.08–0.73). Interestingly, the inconsistency in findings across these two analytic approaches is consistent with the results reported by McColl et al. [25], who found that a continuous measure of UC symptom activity was more strongly correlated with IBDQ scores than with SF-36 scores, but that the IBDQ was not better than the SF-36 at discriminating patients classified by disease extent.

While we expected all instruments to show improvement over the course of treatment in the acute phase, particularly given that improvement in SIBDQ and SF-12v2 in this trial was previously established [19, 22], our sixth hypothesis was that the SIBDQ would exhibit relatively greater sensitivity to treatment than the SF-12v2 since, as a disease-specific measure, the SIBDQ should more precisely capture the differences in HRQL related to treatment for UC symptoms and their improvement as a result of treatment. The data were generally supportive of this hypothesis: The mean effect size for standardized change in domain scores from baseline to endpoint was considerably larger for the SIBDQ (average *d*_*z*_ = 0.62, 95 % CI 0.35–0.89) than for the SF-12v2 (average *d*_*z*_ = 0.33, 0.27–0.39).

While all three instruments showed generally moderate levels of correspondence, each instrument provides a unique and important contribution to understanding the impact of UC, and the effect of treatment for UC, on patients’ lives. The SIBDQ, as would be expected for a disease-specific measure, exhibited moderate-to-high responsiveness to disease activity, thus providing a reliable measure of treatment impact. The SF-12v2 showed moderate responsiveness to disease activity, and as a generic measure that is widely used across many studies and disease areas, it provides the opportunity for a contextual interpretation of the HRQL of patients with UC by facilitating comparisons with other disease samples and general population norms to understand the burden of UC and the degree to which this burden can be relieved through treatment. The WPAI:SHP, with the exception of the absenteeism domain, which was also moderately responsive to changes in disease activity, allows for the most instrumental interpretation of the impact of UC on patients’ lives.

## Conclusion

The findings of mostly moderate correlations among scores on the SF-12v2, SIBDQ, and WPAI:SHP, and between each of these instruments and clinical symptoms, as well as parallel responses to acute and maintenance MMX mesalamine daily treatment, indicate the consistency and correspondence of these instruments within this UC patient population. The finding that all three of these instruments demonstrated sensitivity to treatment and responsiveness to disease activity, with some predictable variations, and the fact that the types of outcomes captured by the instruments are complementary in terms of the interpretation they afford indicate that it is appropriate and beneficial to administer all three of these instruments (or any combination of these instruments depending upon the objectives of the study) for the purpose of capturing the burden of UC and the impact of treatment on quality of life and/or work-related activities in clinical and outcomes research.

## Abbreviations

ANCOVA: Analysis of covariance
ANOVA: Analysis of variance
BP: Bodily pain domain of the SF-12v2
BS: Bowel symptoms domain of the SIBDQ
EF: Emotional functioning domain of the SIBDQ
GH: General health domain of the SF-12v2
HRQL: Health-related quality of life
IBD: Inflammatory bowel disease
IBDQ: Inflammatory Bowel Disease Questionnaire
MH: Mental health domain of the SF-12v2
MMX: Multi Matrix System
PF: Physical functioning domain of the SF-12v2
PRO: Patient-reported outcome
RBS: Rectal bleeding severity
RE: Role emotional domain of the SF-12v2
RP: Role physical domain of the SF-12v2
SF: Social functioning domain of the SF-12v2
SF-12v2: 12-Item Short-Form Health Survey, version 2
SF-36: 36-Item Short-Form Health Survey
SIBDQ: Shortened Version of the Inflammatory Bowel Disease Questionnaire
SIMPLE: Strategies in Maintenance for Patients Receiving Long-term Therapy study
SS: Systemic symptoms domain of the SIBDQ
STF: Stool frequency
UC: Ulcerative colitis
WPAI: Work Productivity and Activity Impairment Questionnaire
WPAI:SHP: Work Productivity and Activity Impairment Questionnaire: Specific Health Problem
WRO: Work-related outcomes
VT: Vitality domain of the SF-12v2

## Footnotes

1 In the SIMPLE trial, quiescence was defined as no more than 1 additional bowel movement than normal and no rectal bleeding.

2 Scores were derived from multiple responses rather than from a single response to reduce error and thus increase precision of scores.

3 Cohen’s *d* effect sizes [45] were calculated using the following equation: $d = 2*f = \sqrt {\eta^{2} /(1 - \eta^{2} )}$, where $\eta^{2} = \left( {df_{\text{between - subjects}} *F} \right)/\left( {df_{\text{between - subjects}} *F + df_{\text{error}} } \right),$ and where *F* is derived from between-subjects ANCOVA with recurrent status as an independent factor, and age, gender, BMI, and maintenance baseline scale value as covariates.

4 Cohen’s *d*_*z*_ effect sizes [45] were calculated using the following equation:
$d_{z} = (M_{1} - M_{2} )/\sigma_{12} = \sqrt {(\sigma_{1} )^{2} + (\sigma_{2} )^{2} {-}[2*r_{12} *\sigma_{1} *\sigma_{2} ]} ,$ with $r_{12}$representing the correlation between scores at each time.
